# Methodological issues on “challenges and opportunities towards the road of universal health coverage (UHC) in Nepal: a systematic review”

**DOI:** 10.1186/s13690-020-00417-y

**Published:** 2020-04-29

**Authors:** Mehrdad Amir-Behghadami, Ali Janati, Masoumeh Gholizadeh

**Affiliations:** 1 Tabriz Health Services Management Research Center, Health Management and Safety Promotion Research Institute, Tabriz University of Medical Sciences, Tabriz, Iran; 2 Iranian Center of Excellence in Health Management, Department of Health Service Management, School of Management and Medical Informatics, Tabriz University of Medical Sciences, University Rd, Golbad, EAZN 5165665811, Tabriz, East Azerbaijan, Iran; 3 Student Research Committee (SRC), Tabriz University of Medical Sciences, Tabriz, Iran

**Keywords:** Systematic review, Methodology, Critical appraisal, Methodological issues, Quality assessment, Data synthesis

## Abstract

Systematic reviews adhere to the principle that science is cumulative and attempt to identify all empirical evidence in accordance with pre-determined eligibility criteria to answer a specific research question. Therefore, in order to achieve reliable findings, these studies must use an explicit method, as they are increasingly used to guide political decision and the direction of future research. We would like to thank the authors Chhabi Lal Ranabhat et al., for the article “Challenges and opportunities towards the road of universal health coverage (UHC) in Nepal: a systematic review”. Although the authors have stated that they reported th according to the Preferred Reporting Items for Systematic Review and Meta-Analysis (PRISMA) guidelines, some items have not been well reported. We critically appraised it using the PRISMA guidelines. Results of the study were significantly valuable, but some important points that hamper the utility of the study need to be considered by the audors. The purpose of this letter is to improve the quality of study and present methodological issues about the search strategy, quality assessment of included studies, and data analysis and synthesis.

To the editor,

We read with great interest the publication entitled, “Challenges and opportunities towards the road of universal health coverage (UHC) in Nepal: a systematic review ”, by Chhabi Lal Ranabhat et al. [1] in the journal, BMC Archives of Public Health. Although the authors have stated that they reported the according to the Preferred Reporting Items for Systematic Review and Meta-Analysis (PRISMA) guidelines, some items have not been well reported. Therefore, the aim of this letter is to present methodological issues about the search strategy, quality assessment of included studies, and data analysis and synthesis.

First, the number of databases searched for literature has been limited to PubMed, the search strategy seems simple, and the only language of interest is English. However, other databases could be searched. This may increase the likelihood of a search bias, i.e. missing some studies language bias, and publication bias. Generally, a systematic review must search major medical databases including PubMed, Scopus, Web of Science, EMBASE and Cochrane Library [2]. Furthermore, in order to identify all eligible studies, each aspect of the research question must be clearly defined in the eligibility criteria. However, inclusion and exclusion criteria have not been fully defined in detail. Running a systematic review without complete knowledge of the inclusion criteria can lead to problems in evaluating the validity, applicability and completeness of the systematic review. Depending on their research questions, the authors could use the components of Population, Intervention, Comparison, Outcomes, and Study design (PICOS) [3].

Second, the authors have ignored the principal component of a full systematic review and have not report the quality of the included studies. Analyzing and interpreting preliminary studies in a systematic review needs qualitative evaluation because poor quality studies can distort the results of the study and affect the quality of the results [4]. The included studies should be evaluated by tools that are appropriate to the study design. If the authors had considered a wide range of types of publications, JBI Critical Appraisal Checklists could have been appropriate because the JBI Scientific Committee had designed specific checklists for all types of studies [4]. If the authors consider studies with quantitative, qualitative, and mixed methods in their inclusion criteria, the use of the Mixed Methods Appraisal Tool (MMAT) would be appropriate [5, 6]. In the MMAT tool, the initial evaluation of each study is performed using two screening questions. If the answer to both questions is “Yes”, the study should be considered for further evaluation. The total quality score for each study is calculated based on the MMAT Scoring Guide [6].

Third, data regarding challenges and opportunities towards the road of UHC data had to been analyzed using a content analysis approach, however the authors have provided no explanation as to what kind of method they used for data synthesis. These data must be categorized in accordance with explicit coding rules and by inductive reasoning into the main category [7].

Systematic reviews are different and more valid than other literature reviews because they provide the best evidence available to researchers. This systematic review should provide an explicit and repeatable methodology. Therefore, it is recommended that physicians, researchers, and journals follow the PRISMA guidelines as it improves the quality of reports of such studies.

## Data Availability

Not applicable.

## Abbreviations

UHC: Universal Health Coverage
PRISMA: Preferred Reporting Items for Systematic Review and Meta-Analysis
PICOS: Population, Intervention, Comparison, Outcomes, and Study design
JBI: Joanna Briggs Institute
MMAT: Mixed Methods Appraisal Tool

## References

[1] Ranabhat CL, Kim CB, Singh A, Acharya D, Pathak K, Sharma B, Mishra SR (2019). Challenges and opportunities towards the road of universal health coverage (UHC) in Nepal: a systematic review. Arch Public Health.

[2] Aromataris E, Riitano D (2014). Systematic reviews: constructing a search strategy and searching for evidence. AJN Am J Nurs.

[3] Amir Behghadami M, Janati A. Population, Intervention, Comparison, Outcomes and Study (PICOS) design as a framework to formulate eligibility criteria in systematic reviews. Emerg Med J. 2020. 10.1136/emermed-2020-209567. 10.1136/emermed-2020-209567 32253195

[4] Porritt K, Gomersall J, Lockwood C (2014). JBI's systematic reviews: study selection and critical appraisal. Am J Nurs.

[5] Amir Behghadami M, Janati A, Sadeghi-Bazargani H, Gholizadeh M, Rahmani F, Arab-Zozani M (2019). Assessing preparedness of non-hospital health centers to provide primary emergency care; A Systematic Review. Bull Emerg Trauma.

[6] Amir Behghadami M, Janati A, Sadeghi-Bazargani H, et al Developing and validating an instrument to assess non-hospital health centers’ preparedness to provide initial emergency care: a study protocol. BMJ Open 2019;9:e026651. 10.1136/bmjopen-2018-026651. 10.1136/bmjopen-2018-026651 PMC6661924 31352412

[7] Hsieh H-F, Shannon SE (2005). Three approaches to qualitative content analysis. Qual Health Res.

